# In vitro growth and carbon utilization of the green-leaved orchid *Dendrobium officinale* are promoted by mycorrhizal associations

**DOI:** 10.1186/1999-3110-54-23

**Published:** 2013-08-28

**Authors:** Qiu-Xia Wang, Ning Yan, Da-Gan Ji, Shu-Yun Li, Hong Hu

**Affiliations:** 1 Key Laboratory of Economic Plants and Biotechnology, Kunming Institute of Botany, Chinese Academy of Sciences, Kunming, 650201 China; 2 University of Chinese Academy of Sciences, Beijing, 100049 China

**Keywords:** ^13^C, *Dendrobium officinale*, Growth, Mycorrhizal associations, Polysaccharides

## Abstract

**Background:**

Mycorrhizal associations play a key role in the life cycle and evolutionary history of orchids. All orchids grow from extremely small seeds that are lacking in reserves, and germination and growth into an underground heterotrophic, achlorophyllous stage depend upon symbiotic fungi to provide nutrient. However, the nutritional physiology between this symbiosis and green-leaved orchids is still unclear. To understand further how these associations affect growth and carbon utilization of green orchids, the green orchids were inoculated with two symbiotic fungi isolated from the roots of a wild orchid (*Dendrobium officinale*) *in vitro* and ^13^C stable isotope signature experiments were designed to analyze carbon nutrition acquisition.

**Results:**

After two months, both fungi had formed mycorrhizal associations with the host roots. Moreover, the growth rate was more rapid for the mycorrhizal seedlings than for the non-mycorrhizal seedlings. The mycorrhizal seedlings not only absorbed more ^13^C from the substrate, but also the S3-mycorrhizal seedlings assimilated more atmospheric ^13^CO_2_ due to significantly higher effective quantum yield of photosystem II, compared with the non-mycorrhizal seedlings. These results suggested that the green orchids could receive more C nutrition from the substrate due to symbiotic fungi, and photosynthesis capacity of the green *D*. *officinale* could be enhanced by the S3 fungus, therefore carbon nutrition acquisition also increased. As a result, the S1- and S3- mycorrhizal seedlings showed markedly higher biomass and polysaccharides contents than the non-mycorrhizal seedlings.

**Conclusions:**

These results improve our understanding of the mycorrhizal functioning in the green *Dendrobium* and show some potential application in the cultivation of *D*. *officinale*.

**Electronic supplementary material:**

The online version of this article (doi:10.1186/1999-3110-54-23) contains supplementary material, which is available to authorized users.

## Background

Mycorrhizal associations have an essential role in the life cycle and evolutionary history of orchids (Rasmussen and Rasmussen 2009). A division of orchids into three physiological types (fully autotrophic, fully mycoheterotrophic (MH), partial MH or mixotrophs) is based on carbon nutrition (Dearnaley et al. 2012). Regardless of their carbon at adult stage, all orchids grow from extremely small seeds that are lacking in reserves, and germination and growth into an underground heterotrophic, achlorophyllous stage (protocorm stage) depend upon symbiotic fungi to provide carbon nutrient (Smith and Read 1997; Arditti and Ghani 2000; Johnson et al. 2007). For adult photosynthetic stage, carbohydrates are also essential for orchid growth (Wang et al., 2008). Moreover, adult orchid roots are consistently heavily colonized by mycorrhizal fungi (McCormick et al. 2006). However, for adult orchids, it is still a matter of discussion whether and to what extent such carbohydrates are actually influenced by their symbiotic fungi. Therefore, accurate measurements of C-source uptake by mycorrhizal and non-mycorrhizal orchids are a critical component to better understanding of mycorrhizal functioning (Cameron et al. 2008).

Stable isotope signatures have proven that some fully autotrophic terrestrial orchids can acquire carbon, nitrogen, phosphate via the “up-flow” pathway through their symbiotic fungi (Cameron et al. 2006,2007,2008; Hynson et al. 2012). Standard experiments with those signatures have been conducted with media containing labeled substrates. C, N and P sources can be supplied through the media, but also carbon can also be obtained through atmospheric CO_2_. The conventional view is that plants can receive C from photosynthate by fixing CO_2_ and the photosynthate can also affect the growth and development in a plant. However, whether the ability of CO_2_ assimilation for green orchids is influenced by its fungal partner still needs experimental confirmation.

A ‘multifunctional’ perspective of mycorrhizal symbiosis accounts for the ways in which such fungi influence carbon-partitioning within plants (Finlay 2004). The concept of carbon nutrition has relied, to a large degree, on studies of carbonhydrates, which, in many cases, are used as a synonym for C accumulation (Mengel and Kirkby 2001). Carbon that is fixed from the atmosphere takes the form of sugars that are, on average, slightly enriched in ^13^C compared with secondary metabolites and structural C (Hynson et al. 2012). Polysaccharides play a vital role in plant metabolism and growth. (Chang and Chou 2007) have reported that mycorrhizae markedly enhance the amount of polysaccharides produced in *Anoectochilus formosanus*. However, the mechanism by which this is accomplished remains unclear. Based on this knowledge, it can be hypothesized that 1) the photosynthetic capacity in green-leaved orchid would improve by their mycorrhizal fungi, and 2) carbon acquisition, soluble polysaccharides accumulation and biomass production would increase in mycorrhizal seedlings.

Here, we provided ^13^CO_2_ and ^13^C-glucose in a quantitative investigation of C-source uptake by both mycorrhizal and non-mycorrhizal orchid plants and determined chlorophyll fluorescence of mycorrhizal and non-mycorrhizal orchid leaves. *Dendrobium* is one of the largest genera in Orchidaceae (Mohanty et al. 2012). Being rich in polysaccharides, *D*. *officinale* is a valuable Chinese traditional medicine that has immunological activity and can inhibit the growth of tumor cells (Luo et al. 2000; Lin et al. 2011). Therefore, positive results from our experiments would be beneficial for its commercial production.

## Methods

### Plant material

#### Adult plants

Wild plants of *Dendrobium officinale* were collected from a subtropical forest at Xishuangbanna (21.7°N, 100.8°E), Yunnan Province, China.

#### Seedlings

Ripe capsules of *D*. *officinale*, collected from a nursery in Puer (22.78°N, 100.97°E), Yunnan Province, China, were washed under tap water and surface-sterilized with 75% ethanol. They were soaked in a 0.1% HgCl_2_ solution for 10 min and then rinsed with sterile distilled water three times. Afterward, they were blotted with sterile filter paper and split. The seeds were sown into culture bottles (8-cm diam) containing 100 mL of a Harvais medium (Harvais 1982) (autoclaved beforehand at 121°C for 30 min) and incubated in the tissue culture room (12 h of daylight, 50 μmol m^-2^ s^-1^, 26 ± 1°C). Germinated seedlings with roots were aseptically transplanted into fresh culture bottles with Harvais medium.

### Isolation of endophytic fungi

Healthy roots from wild plants of *D*. *officinale* were selected, rinsed under tap water, and washed again in sterile distilled water. Once segmented, they were surface-sterilized by consecutive immersions for 8 to 10 min in 0.1% HgCl_2_, and then rinsed five times with sterile distilled water. After surface-drying, the root segments were aseptically cut into approximately 0.5- to 1-cm sections, and transferred to 9-cm Petri dishes containing potato dextrose agar (PDA: 20% potato, 2% glucose, and 1.5% agar). The dishes were incubated in the dark at 25°C until fungal hyphae emerged from inside the roots. Pure cultures were obtained by transferring the hyphae onto fresh PDA and storing them in PDA slant tubes at 4°C. In all of these isolates, two fungi (named S1 and S3) were found to stimulate the growth of tissue-cultured seedlings of *D*. *officinale* through artificial inoculation, so they were selected for further study.

### Molecular identification of fungi

Isolates S1 and S3 were cultured on PDA media. Because other reproductive structures failed to sporulate on that media, we subjected S1 and S3 to molecular analysis of the internal transcribed region (ITS) of the 5.8SrDNA and the large subunit gene of mitochondrial rDNA (mtLSU) sequences, respectively, using universal fungal primer combinations ITS1/ITS4 (Ma et al. 2003) and ML5/ML6 (Bruns et al. 1998). DNA was extracted from 30-day-old PDA-cultured colonies according to the cetyltrimethyl ammonium bromide (CTAB) method (Doyle and Doyle 1987). PCR reactions (25 μL) were performed using 2.5 μL of 10 × buffer (with Mg^2+^), 2 μL of 2.5 mM dNTP, 0.5 μL of TaqE (2.5U), 2 μL of 5 μM of each primer, 2 μL of undiluted DNA template and 14 μL of ddH_2_O. The cycle parameters included denaturation at 95°C for 3 min; then 35 cycles of denaturation at 94°C for 1 min, annealing at 53°C for 50s, and elongation at 72°C for 1 min; followed by a final extension at 72°C for 7 min. The PCR products were purified and directly sequenced in an ABI Prism 3730 Sequencer (Applied Biosystems, Foster City, CA, USA) at the Shanghai Sangon Biological Engineering Technology & Services Co. Ltd. The sequences were aligned by Contig Express and adjusted manually.

### Inocula of fungi

Samples of the S1 and S3 fungi were transferred to 9-cm Petri dishes containing PDA media for incubation in the dark at 25°C for three weeks, respectively.

### Inoculation experiments

Before experimental analysis, the two-month-old seedlings of *D*. *officinale* were trimmed to provide uniformly sized materials, each consisting of a stem supporting three to five leaves and two to three roots of 20 mm in length. Eight uniform seedlings were implanted into one culture bottle containing 100 mL of 1/2 MS (half-strength concentrations of the major and minor salts of the MS medium) (Murashige and Skoog 1962) medium supplemented with 0.75% sucrose and 0.75% agar after weighed (Hou and Guo 2009). For the inoculation treatments, a single mycelia plug (6-mm diam), cut from the margin of a fungal colony (either S1 or S3), was placed into the middle of each bottle. The control involved eight similarly placed seedlings that were not inoculated with any fungi. Each treatment comprised 25 replicates. All treatments were kept in the tissue culture room for two months.

### Visualization of fungal infection in roots

The fungal hyphae were stained with a chitin-specific dye, wheat germ agglutinin-alexa fluor (WGA-AF) 488 conjugate (Molecular Probes, Karlsruhe, Germany). Fungal infection was observed through a previously described technique (Doehlemann et al. 2008) with some modifications. After inoculation period, the root segments were incubated in the staining solution 10 μg/ml WGA–AF 488; 0.02% Tween 20 in 1× PBS (137 mM NaCl, 27 mM KCl, 100 mM Na_2_HPO_4_, 2 mM K_2_HPO_4_, adjusted to pH 7.4) for 60 min; during this period, the solution was vacuum infiltrated three times (3 min each) at 25 mm of Hg. After the samples were rinsed in 1 × PBS (phosphate buffer saline) (pH 7.4) three times, they were transferred into a Propidium Iodide (PI, Sigma) solution (20 μg mL^-1^ PI in 1× PBS) for 3 min and then rinsed in 1 × PBS (pH 7.4) three times. Finally, the samples were mounted on glass slides.

Confocal images were recorded on a laser scanning confocal microscope (OLYMPUS FV1000). The WGA-AF 488 was excited with a 488 nm laser and detected at 500 to 540 nm. The PI was excited with a 559 nm laser and detected at 580 to 619 nm.

### Light microscopy examination

Twenty root segments were fixed in anhydrous ethanol for above 24 h, and then freehand sections were observed and photographed on a light microscope equipped with a camera (Leica DM 2500, Germany).

### Chlorophyll fluorescence measurements

We conducted measurement for the light response of chlorophyll fluorescence in leaves from in vitro grown seedlings at 25°C with IMAGING-PAM (Heinz Walz, Effeltrich, Germany) connected to a computer with control software. Six mature leaves from eight seedlings of each treatment were light-adapted (55 μmol photons m^-2^ s^-1^) for 15 min at 25°C before the measurement of light response curves. The effective quantum yield of PSII (Φ_PSII_) is calculated as: Φ_PSII_ = (*F*_*m*_’ - *F*_*s*_)/*F*_*m*_’ (Genty et al. 1989), where *F*_*m*_’ is the maximum fluorescence values upon illumination of a pulse (800 ms) of saturating light (10,000 μmol photons m^-2^ s^-1^) in the light-adapted state, and *F*_*s*_ is steady-state fluorescence in light.

### Experimental design for ^13^C stable isotope signature in a microcosm

**Figure 1:**
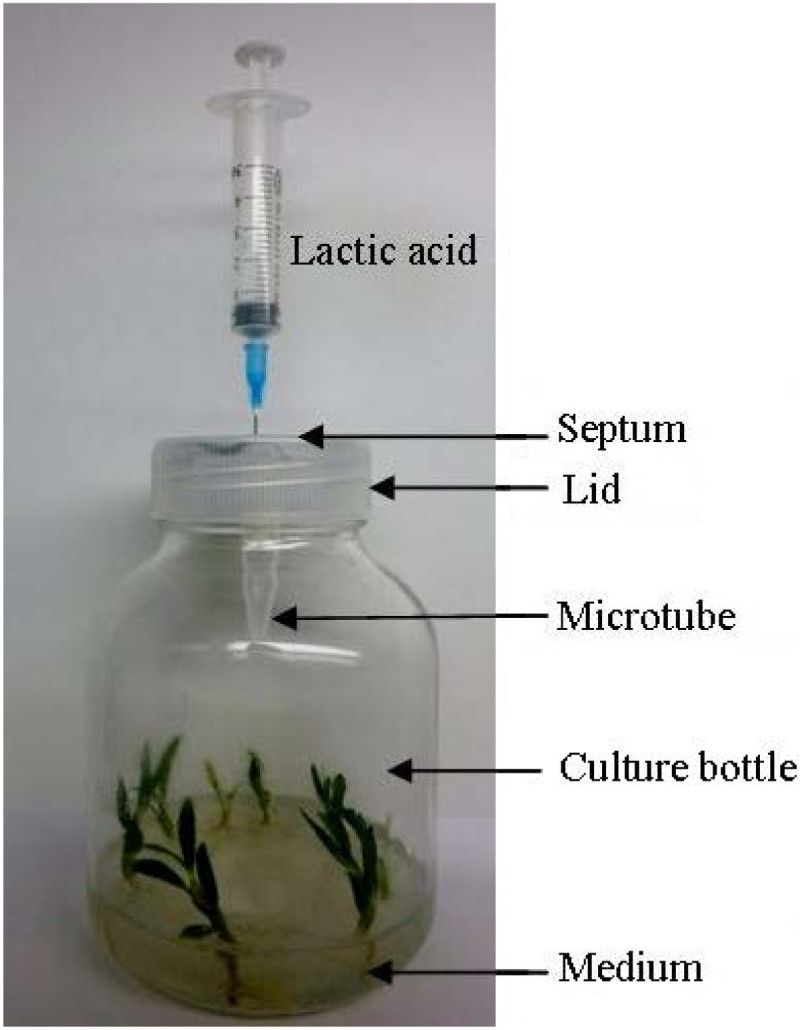
**Culture-bottle microcosm used to supply isotopically labelled CO**_**2**_**and glucose to the orchid seedlings.** Lactic acid was injected into the microtube with Ba^13^CO_3_ to liberate ^13^CO_2_ through septum in lid. Medium contained ^13^C-labelled glucose.

Each of our experimental microcosms consisted of a culture bottle with a round, sealed septum (8-mm diam) passed through the middle of the lid (Figure 1). To supply ^13^CO_2_, each microcosm contained a 1.5-mL microtube with 30 mg of sterile ^13^C-labelled barium carbonate, plus 100 mL of 1/2 MS medium supplemented with 100 mg of unlabelled glucose and 0.75% agar. To determine C absorbed from substrates by plants, each microcosm had a 1.5-mL microtube with 30 mg sterile unlabelled barium carbonate and 100 mL of 1/2 MS medium supplemented with 100 mg of ^13^C-labelled glucose and 0.75% agar. Before the experiments began, the seedlings were trimmed to provide uniformly sized materials, each having three to five leaves and two to three roots. Three treatments per labeling experiment were established, with each treatment prepared as described for our inoculation experiments. However, a mycelia plug was placed near the roots of each seedling, rather than in the middle of medium, so that fungal infection would occur as soon as possible. Replicate treatments (n = 6) were preformed in case of contamination. All treatments were placed into the tissue culture room (12 h of daylight, 50 μmol m^-2^ s^-1^, 26 ± 1°C). To liberate the ^13^CO_2_ and unlabelled CO_2_ from the ^13^C-labelled and unlabelled barium carbonate, respectively, we injected 0.2 mL of a 25% (v: v) sterile lactic acid solution injected through the sealed septum in the middle of each lid. Afterward, the microcosms were sealed until the end of the experimental period.

### Analysis of ^13^C stable isotope abundance

After two months, the plants were harvested from each bottle and dried at 80°C for 48 h. They were then ground to a fine powder to obtain a representative sub-sample of plant tissue for further analysis. Relative C isotope abundances were measured by isotope ratio mass spectrometer (Delta V Advantage, USA) at Institute of Desertification Studies, China Academy of Forestry, as described by (Liebel and Gebauer 2011). Three test substances of varying sample weight were routinely analyzed within each treatment. The maximum variation in δ^13^C was always below 0.1%.

### Determination of polysaccharides contents

Dry samples from the aboveground parts of each treatment were homogenized, and their percentages of polysaccharides were determined by the phenol-sulphuric acid method (Pharmacopoeia Committee of the P.R. China 2010). Each treatment was performed in triplicates to minimize errors.

### Data collection and statistical analysis

Two months after inoculation, the plants were harvested to record their heights, stem lengths, stem diameters, internode lengths and numbers of nodes. For each culture bottle, the fresh and dry weights, and the numbers of new roots and tillers also were determined. The eight plants from each bottle were weighed and then dried for 48 h at 80°C before measuring dry weight. The increment in fresh weight per bottle was calculated as plants weight after inoculation minus plant weight before inoculation. The statistical package SPSS 17.0 for Windows was used for data analysis. Differences among the treatments and control groups were tested with a one-way analysis of variance (ANOVA), followed by tests for least significant difference test. Values were presented as means ± standard error (SE).

## Results

### Formation of mycorrhizal associations

**Figure 2:**
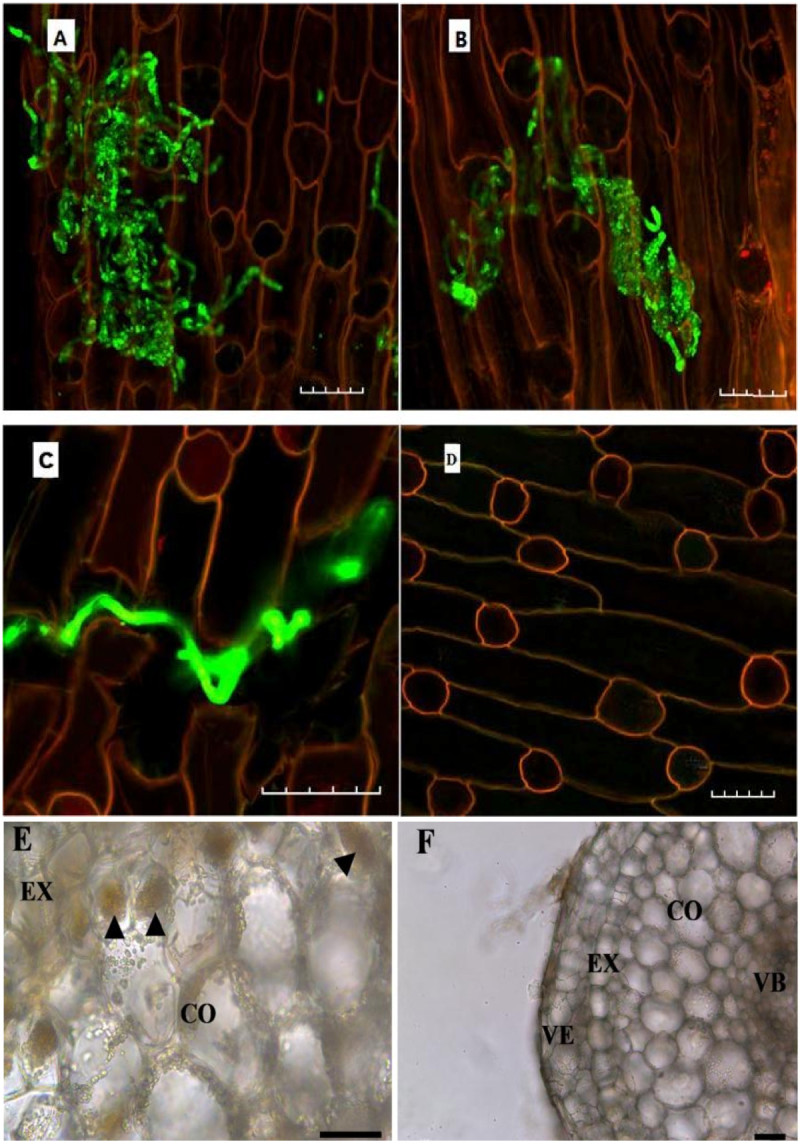
**Mycorrhizal associations of** ***Dendrobium officinale*** **roots. A**: Hyphae (green) from S1 fungus colonizing root cortical cells; **B**: Hyphae (green) of S3 fungus colonizing root cortical cells; **C**: Hyphae (green) penetrating cell wall and invading a neighbor cell in the root; **D**: Root cortical cells from non-inoculated control seedlings; **E**: Transverse section showing pelotons formed in the cortical cells of the inoculated roots; **F**: Transverse section of the control root. *arrows*, peloton; VE, velamen; EX, exodermis; CO, cortex; VB, vascular bundle Scale bar = 50 μm.

After two months of inoculation, S1 and S3 hyphae had spread over the root surfaces, invaded the cortical cells, and colonized the intracellular spaces of the roots of the inoculated seedlings (Figure 2A, B, E). In the cortical region, the hyphae penetrated through the cell walls and entered next to the cortical cells (Figure 2C). No fungal hyphae or pelotons were observed within the root cells from the control (non-inoculated) seedlings (Figure 2D, F).

### Growth responses of *D*. *officinale*

Two months after being inoculated with S1 or S3, the host plants showed enhanced growth (Table 1). Compared with the control seedlings, increments of fresh weight, dry weight, plant heights, stem lengths and diameters, internode lengths, and number of nodes, tillers and new roots were significantly higher in the mycorrhizal seedlings.

**Table 1:** **Effects of S1 and S3 on the growth of** ***Dendrobium officinale*** **seedlings (n = 25 each)**

Parameters	S1	S3	Control
Increment in fresh weight (g)	0.96 ± 0.06b	1.27 ± 0.05c	0.57 ± 0.02a
Dry weight (g)	0.06 ± 0.00b	0.09 ± 0.00c	0.03 ± 0.00a
Plant height (cm)	2.16 ± 0.04b	2.12 ± 0.06b	1.81 ± 0.07a
Stem length (cm)	1.01 ± 0.02b	1.14 ± 0.04c	0.89 ± 0.03a
Stem diameter (cm)	0.18 ± 0.01b	0.28 ± 0.01c	0.15 ± 0.01a
Number of nodes	3.09 ± 0.06b	3.22 ± 0.11b	2.72 ± 0.01a
Internode length (cm)	0.31 ± 0.01b	0.33 ± 0.01b	0.25 ± 0.01a
Number of tillers	26.24 ± 0.99c	19.40 ± 0.75b	7.24 ± 0.60a
Number of new roots	21.80 ± 1.45c	15.00 ± 1.00b	9.68 ± 0.68a

Different letters within the same row indicate that values (mean ± standard error) are significantly different among treatments at *P* < 0.05, based on LSD test. Control, non-inoculated seedlings.

The mycorrhizal fungi were re-isolated from the *D*. *officinale* roots after two months of inoculation. As expected, no fungus was isolated from the non-inoculated orchids. We then sequenced the ITS-5.8S rDNA sequences of the re-isolated fungus from the roots inoculated with S1 as well as the mtLSU sequences of the re-isolated fungus from the S3-inoculated roots and verified that these fungi were the same as those used for the first inoculations (data unpublished).

### Chlorophyll fluorescence

**Figure 3:**
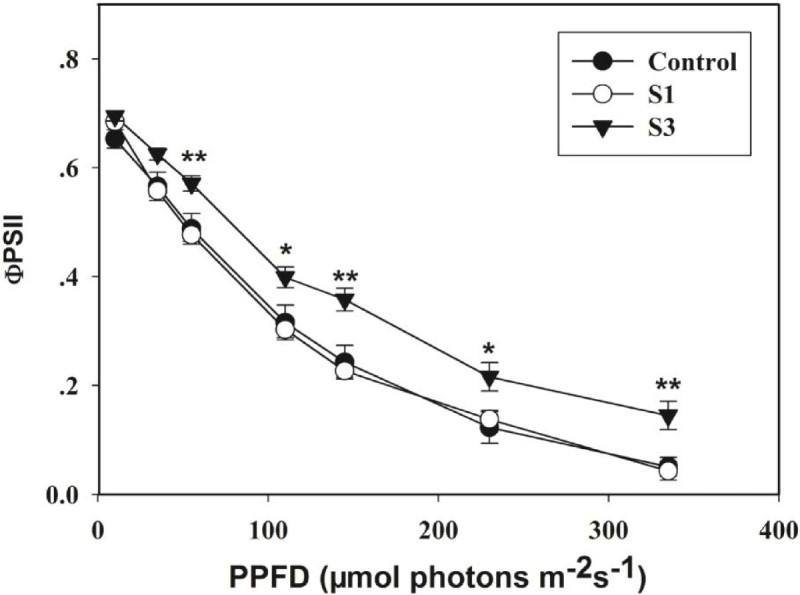
**Responses of ФPSII to incident PPFD for leaves of mycorrhizal and non-mycorrhizal** ***Dendrobium officinale*** **seedlings.** Data are the means of six replicates; SES are denoted by error bars. Single asterisk (*) indicates a significant difference compared with the control at *P* < 0.05; double asterisk (**) indicates a significant difference compared with the control at *P* < 0.01.

Values for ФPSII in the S3-mycorrhizal seedlings were significantly higher than those in the S1-mycorrhizal and non-mycorrhizal seedlings. However, values for ФPSII did not differ between the S1- mycorrhizal and non-mycorrhizal seedlings (Figure 3).

### Carbon utilization by different treatments

**Figure 4:**
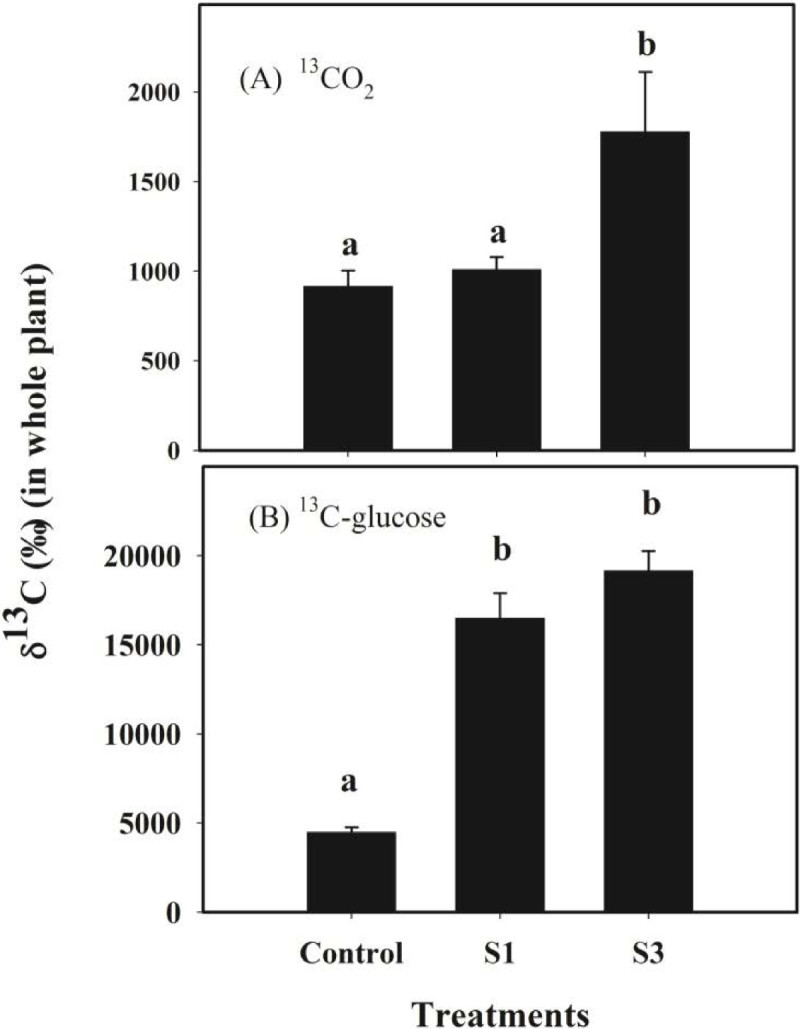
**Whole-plant δ**^**13**^**C measurements from mycorrhizal and non-mycorrhizal seedlings of** ***Dendrobium officinale*** **(n = 3 each).** Values (mean ± standard error) not followed by the same letter are significantly different at *P* < 0.05, based on LSD test. **(a)** derived from ^13^CO_2_; and **(b)** from ^13^C-labelled glucose. Control, non-inoculated seedlings.

Both ^13^C derived from ^13^CO_2_ and ^13^C from ^13^C-labelled glucose were readily detectable in the mycorrhizal and non-mycorrhizal seedlings (Figure 4). For the seedlings fed with only ^13^CO_2_, the δ^13^C value in the S3-mycorrhizal seedlings was about 1.94-fold higher than that in the non-mycorrhizal seedlings, and was also significantly different from that calculated for the S1-mycorrhizal seedlings. However, value for δ^13^C derived from ^13^CO_2_ had no significant difference between the S1-mycorrhizal and non-mycorrhizal seedlings (Figure 4A). By contrast, δ^13^C derived from ^13^C-glucose in the S1- and S3-mycorrhizal seedlings was about 3.67- and 4.34- fold higher, respectively, than that in the control seedlings. Again, however, the δ^13^C value derived from ^13^C-glucose had no significant difference between the S1 and S3 treatments (Figure 4B).

### Polysaccharides contents

**Figure 5:**
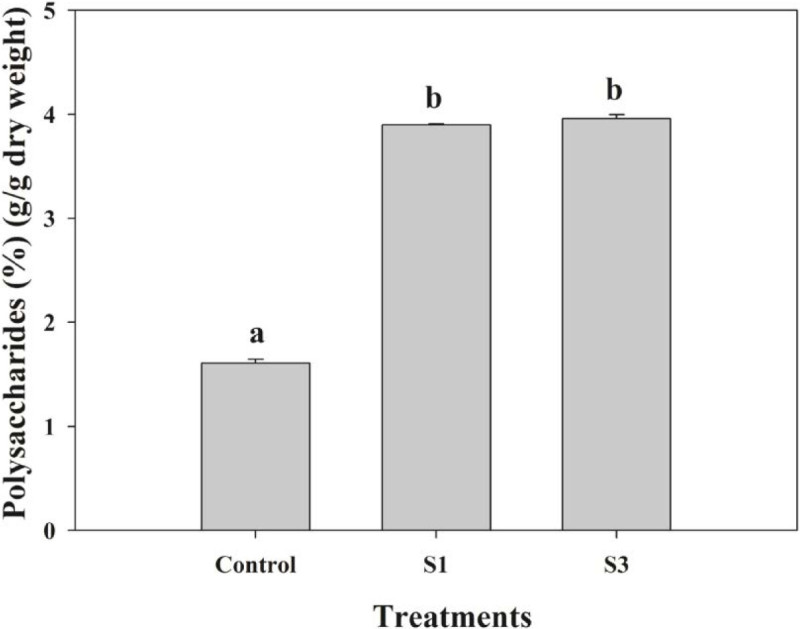
**Effects of S1 and S3 on polysaccharides contents in** ***Dendrobium officinale*** **seedlings (n = 3 each).** Values (mean ± standard error) not followed by the same letter are significantly different at *P* < 0.05, based on LSD test. Control, non-inoculated seedlings.

Compared with the non-mycorrhizal seedlings, the mycorrhizal seedlings had significantly higher polysaccharides percentages when harvested. Contents in the seedlings treated with S1 and S3 fungi were 1.42- and 1.46- fold, respectively, of those measured from the control. However, the two inoculation treatments did not differ significantly in their polysaccharide accumulations (Figure 5).

## Discussion

The functioning of mycorrhizae depends upon the development of an association between a fungus and a host plant (Johnson et al. 1997). We demonstrated here that two isolates formed mycorrhizal associations with *Dendrobium officinale*. Both S1 and S3 markedly enhanced the growth of *D*. *officinale* seedlings. Although it has previously been documented that symbiotic fungi can promote the growth of their host plants (Stewart and Kane 2007; Zhang et al. 2012), the mechanism by which this occurs remains unclear.

Few previous studies have quantified the amount of CO_2_ that is assimilated in mycorrhizal versus non-mycorrhizal plants. Our study suggested that the amount of CO_2_ that is assimilated in the S3-mycorrhizal seedlings was significantly higher than that in the non-mycorrhizal seedlings. Some evidences have been reported that mycorrhizal seedlings have higher stomatal conductance and chlorophyll concentrations than non-mycorrhizal seedlings (Sheng et al. 2008; Zhou et al. 2009). This implies that the mycorrhizal seedlings have greater photosynthetic capacity. Another explanation for this phenomenon might be that a fungus can serve as a nutrient sink, because some orchid plantlets can transfer a substantial amount of photosynthate to its symbiotic fungi (Cameron et al. 2006). Other explanations might be that mycorrhizae can decrease the resistance (stomatal and mesophyll) to CO_2_ diffusion, or increase the activities of carboxylating enzymes, or change hormones levels in mycorrhizal plant (Reid et al. 1983). In the present study, to study photosynthesis we used chlorophyll fluorescence analysis, which has become one of the most powerful and relatively straightforward techniques. The results pointed out that ФPSII in the leaves of the S3-mycorrhizal seedlings was significantly higher than that in the S1-mycorrhizal and non-mycorrhizal seedlings. This implied that the capacity of photosynthetic electron flow of the S3-mycorrhizal seedlings was higher than that of the S1-mycorrhizal and non-mycorrhizal seedlings. Because there is a positive correlation between photosynthetic electron flow and CO_2_ assimilation, the amount of photosynthetic CO_2_ assimilation in the S3-mycorrhizal seedlings was higher than that in the S1-mycorrhizal and non-mycorrhizal seedlings.

Our results indicated that, compared with the non-mycorrhizal seedlings, both of the S1- and S3- mycorrhizal seedlings absorbed more C sources from the substrate. It has been confirmed experimentally that fungal symbiont can transfer C to the adult *Goodyera repens* (Cameron et al. 2006). That is, adult mycorrhizal orchids continue to receive C nutrition from their fungal partners, which is possibly because that the photosynthetic products cannot meet the demand of plant growth and development. Some researchers have found that mycorrhizal fungi could induce significant changes in the morphology of the host plant roots by increasing branches lengths and diameters (Hooker and Black 1995; Zhang et al. 2012). In another study, we also found that the host plant produced a larger number of root hairs (data unpublished). Such responses enable the plant to absorb more nutrients from the substrate. But it needs further investigation that the C enhancement from ^13^C-glucose in our orchid is due to direct absorption of host roots or transferring from intraradical fungal structure.

In addition, we found that the S1- and S3- mycorrhizal seedlings had significantly higher polysaccharides contents. It has been widely recognized that, during photosynthesis, atmospheric CO_2_ can be fixed to produce carbohydrates, especially polysaccharides. Our results demonstrated that the S3-mycorrhizal seedlings assimilated a larger amount CO_2_, indicating that more carbohydrates (including polysaccharides) were being accumulated. Moreover, soluble sugars within the substrate, e.g., sucrose and glucose, are effectively taken up after being transferred to the roots via the associated fungi (Pfeffer et al. 1999; Hynson et al. 2012). And soluble sugars are the common precursors of polysaccharides synthesis (Zha et al. 2007). Our results indicated that the mycorrhizal seedlings absorbed significantly more C from glucose than the non-mycorrhizal seedlings. Hence, we suggested that the increase of polysaccharides content in mycorrhizal plants may be related to the increase of C sources.

Plant polysaccharides are classified as either structural or non-structural (Potkins et al. 1991). The structural components of plant cells largely comprise carbohydrates, and are critical to cell wall functioning (Caffall and Mohnen 2009). Non-structural polysaccharides serve as an energy reserve for initial cell growth and expansion (Percival 1979). Thus, in our present study, the enhancement of biomass in the mycorrhizal seedlings was probably due to an increase in their non-stuctural polysaccharides accumulation.

## Conclusions

In this study, two endophytic fungi (S1 and S3) sampled from wild *D*. *officinale* formed *in vitro* mycorrhizal associations with host roots, thereby markedly enhancing seedling growth. Both the S1- and S3- mycorrhizal seedlings received a larger amount of C source from substrates, while those seedlings exposed to the S3 fungus assimilated more CO_2_, which led to a higher percentage of polysaccharides. S1 and S3 differed in their extent of influence on the capacity of photosynthetic electron flow of *D*. *officinale*, indicating that they may have separate roles within the same host. Although we investigated carbon utilization of the green orchid in experimental conditions, further investigations should focus on the effects of symbiotic fungi on carbon nutrient acquisition of the adult *D*. *officinale* under field conditions. Moreover, the mechanism for increased photosynthesis capacity and polysaccharides accumulation via mycorrhizal associations should be explored in a more comprehensive way.

## Authors’ original submitted files for images

Below are the links to the authors’ original submitted files for images. Authors’ original file for figure 1 Authors’ original file for figure 2 Authors’ original file for figure 3 Authors’ original file for figure 4 Authors’ original file for figure 5

## Abbreviations

TAB: Cetyltrimethyl ammonium bromide
ITS: Internal transcribed region
mtLSU: Large subunit gene of mitochondrial rDNA
PBS: Phosphate buffer saline
PDA: Potato dextrose agar
PI: Propidium Iodide
PPFD: Photosynthetic photon flux density
WGA-AF: Wheat germ agglutinin-alexa fluor
ФPSII: Quantum yield of PSII.
