# Supplementary figures and images for: In vitro growth and carbon utilization of the green-leaved orchid Dendrobium officinale are promoted by mycorrhizal associations
Source: Bot Stud. 2013 Aug 28;54:23. doi: 10.1186/1999-3110-54-23 (PMC5430334; doi:10.1186/1999-3110-54-23)

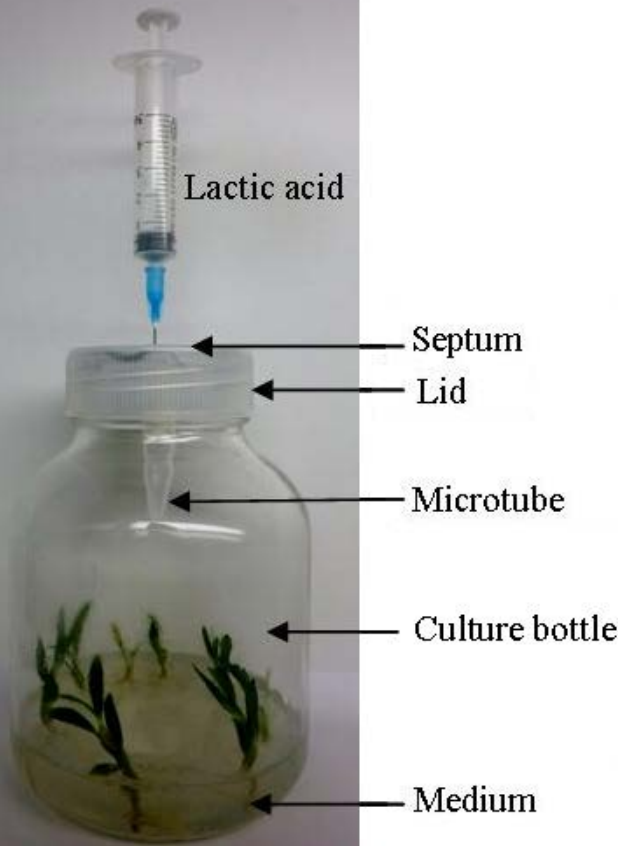

Supplement: Supplementary file 1 — Authors’ original file for figure 1 [file 40529_2013_93_MOESM1_ESM.pdf]

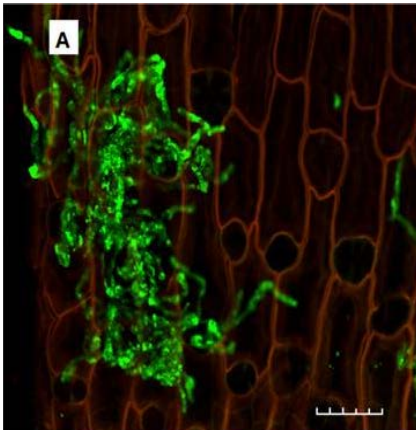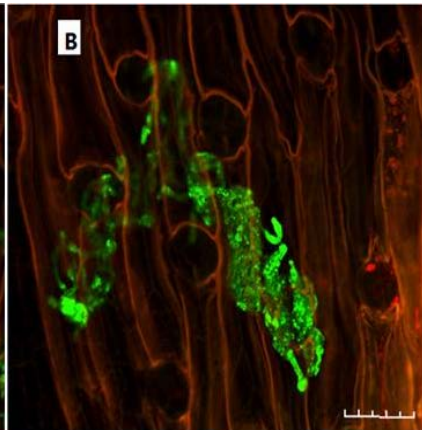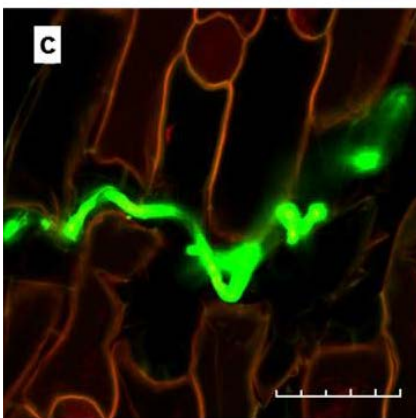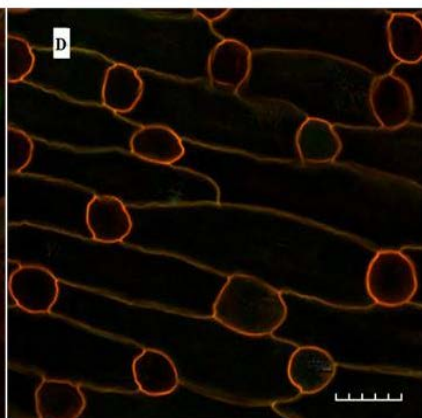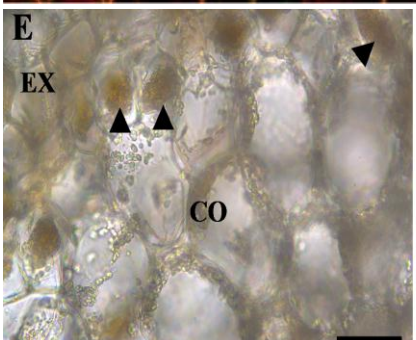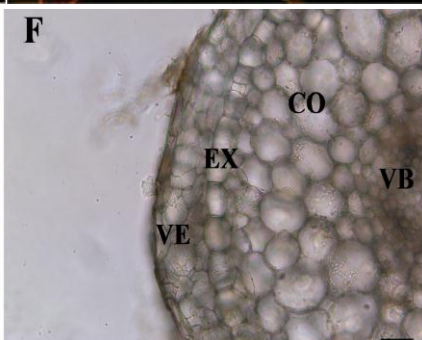

Supplement: Supplementary file 2 — Authors’ original file for figure 2 [file 40529_2013_93_MOESM2_ESM.pdf]

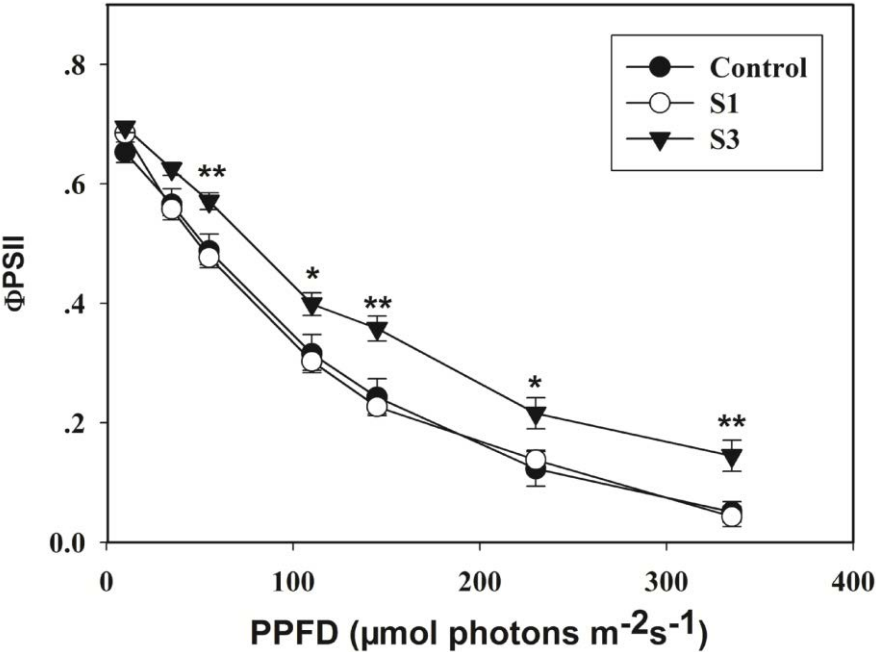

Supplement: Supplementary file 3 — Authors’ original file for figure 3 [file 40529_2013_93_MOESM3_ESM.pdf]

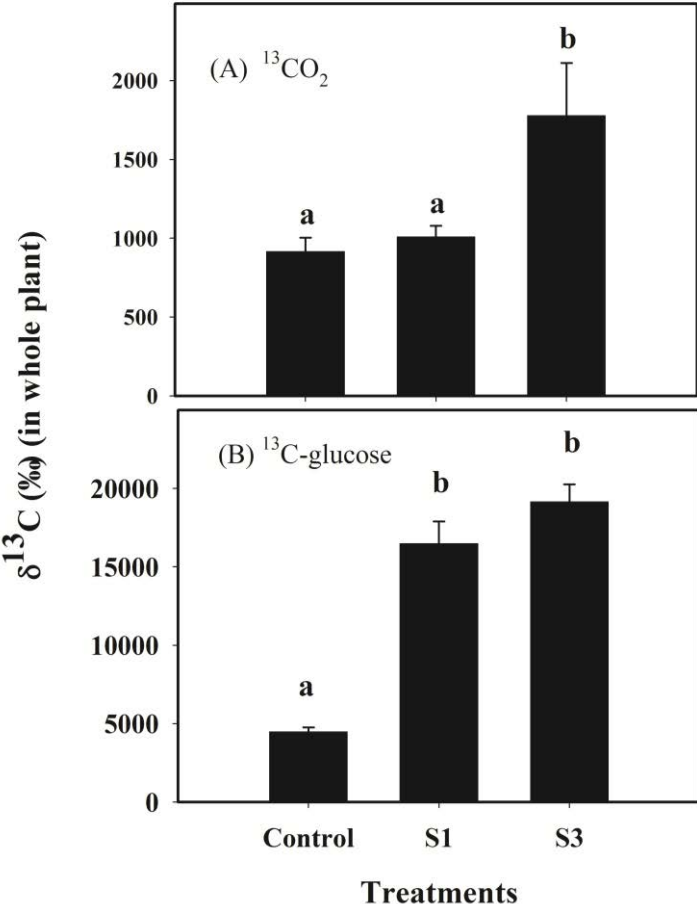

Supplement: Supplementary file 4 — Authors’ original file for figure 4 [file 40529_2013_93_MOESM4_ESM.pdf]

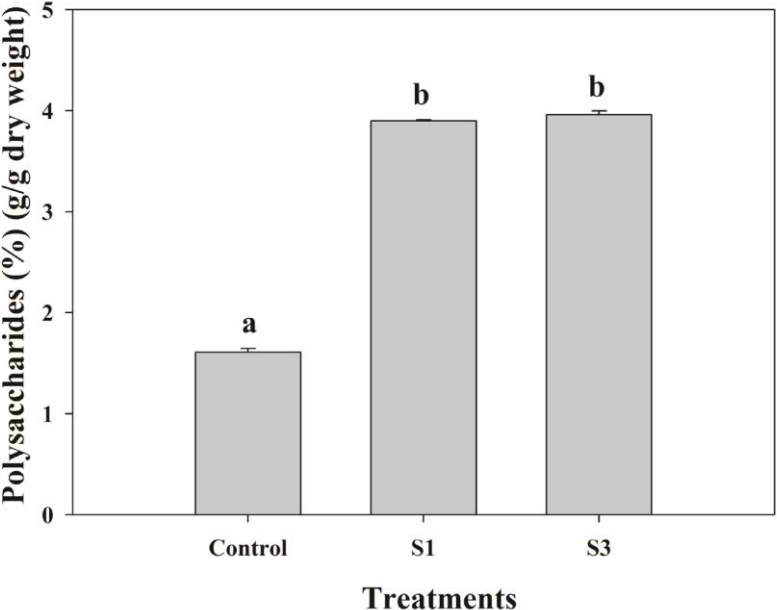

Supplement: Supplementary file 5 — Authors’ original file for figure 5 [file 40529_2013_93_MOESM5_ESM.pdf]
